# Assessment of surface roughness changes on orthodontic acrylic resins by all-in-one spray disinfectant solutions

**DOI:** 10.34172/joddd.2020.019

**Published:** 2020-06-17

**Authors:** Kuei-ling Hsu, Abdulrahman A. Balhaddad, Isadora Martini Garcia, Fabrício Mezzomo Collares, Louis DePaola, Mary Anne Melo

**Affiliations:** ^1^Ph.D. Program in Dental Biomedical Sciences, University of Maryland School of Dentistry, Baltimore, MD 21201, USA; ^2^Division of Pediatric Dentistry, Department of Orthodontics and Pediatric Dentistry, University of Maryland School of Dentistry, Baltimore, MD 21201, USA; ^3^Department of Restorative Dental Sciences, Imam Abdulrahman Bin Faisal University, College of Dentistry, Dammam, Saudi Arabia; ^4^Dental Materials Laboratory, School of Dentistry, Federal University of Rio Grande do Sul, Porto Alegre, 90035-003, RS, Brazil; ^5^Department of Oncology and Diagnostic Sciences, University of Maryland School of Dentistry, Baltimore, MD 21201, USA; ^6^Division of Operative Dentistry, Department of General Dentistry, University of Maryland School of Dentistry, Baltimore, MD 21201, USA

**Keywords:** Acrylic resin, Disinfection, Orthodontic, Surface roughness

## Abstract

**Background.** The disinfection of orthodontic acrylic resins might change the physical and mechanical properties of these materials. We aimed to investigate the impact of four different commercially available disinfectants on the surface roughness of acrylic resins used for orthodontic appliances.

**Methods.** Four disinfectant solutions (BirexSE, Opti-Cide3, COEfect MinuteSpray, and CaviCide Spray) were used to disinfect orthodontic acrylic resins using the spraying method. The resins were subjected to repeated disinfection protocols. Distilled water, also applied via spraying method, was used as a control. Surface roughness was scrutinized to examine the extent of surface topography changes by stylus profilometry. Data normality was evaluated via the Shapiro–Wilk test, followed by the Wilcoxon Signed-Rank test for non-parametric data or paired Student’s t-test for parametric data to compare intra-group differences in roughness before and after the use of the disinfectant solutions.

**Results.** Some of the disinfectants (BirexSE and CaviCide) resulted in significant changes in surface roughness values before and after the disinfection compared to the controls (P<0.05). The groups that were in contact with distilled water, Opti-Cide, and Coeffect did not exhibit significant differences in surface roughness before and after the intervention (P>0.05). However, from a clinical perspective, the resulting variations in surface roughness (<%0.15) induced by these solutions might not reflect clinically significant differences.

**Conclusion.** The use of disinfectant solutions is unlikely to harm the surface of orthodontic acrylic resins. Oral care providers need to be attentive to the interpretation and implementation of clinically significant changes in their evidence-based approach regarding potential material damages by disinfection sprays.

## Introduction

Wearing removable orthodontic appliances to move or retain teeth is part of the orthodontic treatment.^1,2^ Often, these appliances are recommended for long-term or even permanent nocturnal wearing.^3^ Cleaning of removable orthodontic appliances can be achieved by manual brushing and chemical agents.^4^ The cleaning of removable orthodontic appliances reduces plaque accumulation and minimizes the risk of developing dental caries, periodontitis, and fungal infections.^5^ The use of fluoride-containing dentifrices is suggested to prevent biofilm formation over orthodontic appliances.^6^ However, the mechanical control of oral biofilms could be compromised by certain factors such as low-standard products and the frequency of home oral hygiene practice.^7^

Orthodontic appliances are not frequently disinfected or cleaned in routine dental settings.^8^ As there is no specific guideline to eliminate microbial biofilms from the surface of orthodontic appliances, disinfection solutions have been suggested to eliminate oral microbes from critical retentive sites on the appliances.^4^

Solutions applied for intended disinfection should preserve their physical and mechanical properties, not compromising the surface integrity of the material.^9^ The extent of the roughness of an orthodontic appliance can influence the biofilm attachment, which might accelerate the colonization of microorganisms and alter the appliance’s color.^10^ This condition is critical as orthodontic appliances must have a smooth surface to prevent the adhesion and colonization of oral microbes.^11^ Ideally, frequent disinfection of surface material should prevent or modulate bacterial growth by removing the microorganisms entrapped in the microporosities of the acrylic resin surface.^12,13^ Additionally, oral microorganisms should be removed or killed using an effective cleaning method without changing the smooth surface to maintain optimal oral health in orthodontic patients.^14^

The early attachment and retention of microorganisms on acrylic materials depend on the surface free energy and the surface microroughness. Additionally, a low-energy surface might prevent plaque stagnation on acrylic materials.^8^ It is imperative to keep in mind that surface roughness is a more significant influencing factor than the surface free energy for bacterial growth. A polished surface might alter the surface energy of such materials, affecting bacterial adhesion.^15^ Therefore, rougher surfaces can facilitate and increase the rate of bacterial accumulation on acrylic resins.

Microorganisms can adhere firmly to the acrylic resin surface by chemical bonding, leading to biofilm formation and growth.^16^ The attached bacteria and fungi can then intermingle with the resin surface by direct contact to form the biofilm.^17^ As a result, acrylic appliances with a roughness >0.2 µm, the reported threshold, are at higher risk of being colonized by oral microbes.^18^ Based on the above considerations, previous studies have evaluated the impact of disinfectant solutions on the surface roughness of acrylic resins.^19,20^ In this context, the present research evaluated the impact of chemical disinfection on the surface roughness of acrylic resins intended for orthodontic appliance fabrication. This in vitro study hypothesized that different disinfection solutions could adversely affect the extent of the surface roughness of acrylic resins with repeated use of chemical disinfection.

## Methods

### Experimental design

This study investigated the effect of different disinfectant solutions (BirexSE, Opti-Cide3, COEfect MinuteSpray, and CaviCide Spray) on the roughness of acrylic resin used for orthodontic appliances. Table 1 displays a description of each material used in this study.

**Table 1:** The compositions and manufacturers of orthodontic acrylic resin and the one-step spray disinfecting solutions.

**Cleaner**	**Manufacturer**	**Ingredient -Weight %**	**Purpose/ Function**	**Presentation**
**Ortho-Jet Crystal Orthodontic Resin Acrylic Self Cure Clear**	Lang Dental Wheeling, IL, USA	Powder: Polymethylmethacrylate- >90; Liquid: Methyl Methacrylate >95%	Dental monomers	Powder (5 lbs.) and Liquid 500 mL
**BirexSE**	Biotrol, Earth City, MO	Isopropyl alcohol 5-10%; 2-Butoxyethanol 1-5% ; Phosphoric acid 15-17% ; 2-Phenylphenol 5-10%; 4-tert-Pentylphenol 5-10%; sulfonic acids, sodium salts, C14-16 alkane hydroxyl 5-10%; C14-16 alkane 5-10%	Germicidal Germicidal Control pH Germicidal Germicidal Foaming agent, Dry agent Stabilizers Germicidal Germicidal	Powder to be dissolved One 1.8 ounce (3.70 mL) packet to each pre-measure quart (0.946L)
**Opti- Cide**	Micro-Scientific, LLC Gurnee, IL	Isopropyl alcohol 10-30% 2-Butoxyethanol 1-5%	Germicidal Germicidal	24 oz. trigger spray bottle
**COEfect MinuteSpray**	GC America Inc, Alsip, IL	Ethyl alcohol 60-80%	Germicidal	24 oz. trigger spray bottle
**CaviCide Spray**	Metrex Research Corporation, Orange, CA	Isopropanol 10-20%; 2-Butoxyethanol 1-5%; Diisobutylphenoxyethoxyethyldim ethylbenzylammonium chloride 0.28%	Germicidal Germicidal Germicidal	24 oz. trigger spray bottle

### Sample preparation 

An auto-polymerizing commercially available orthodontic acrylic resin (Ortho-Jet; Lang Dental Manufacturing Co. Inc., Wheeling, IL, USA) was used. The proportion of powder to liquid (2:1 ratio), as indicated by the manufacturer, was followed. The mixed acrylic resin was dispersed into 10-mm-diameter stainless steel molds. Each of the 50 cylinders was covered and separated by transparent polyester strips. Following the manufacturer’s instructions, a high-capacity hydraulic-pressure curing unit prepared for use with self-curing resins (Aquapres, Lang Dental Manufacturing Co. Inc., Wheeling, IL, USA) was used to process the molds containing the cylinders. Subsequently, the cured disks were easily detached from molds by extrusion and available for the evaluation of roughness.

### Disinfection procedure

For each set of specimens (n=10), ten sprays of either sterile water (control) or one of the disinfectant solutions were applied and spread throughout the surface area. The treated specimens were exposed to air and immediately dried after the air exposure. Complete simulated disinfection included spraying ten times at 5-second intervals, assuring a one-minute contact time of the disinfectant as stipulated on the label of the disinfectant solutions. The specimens were not polished and subjected to the assessment of surface roughness.

### Assessment of surface roughness 

**Figure 1:**
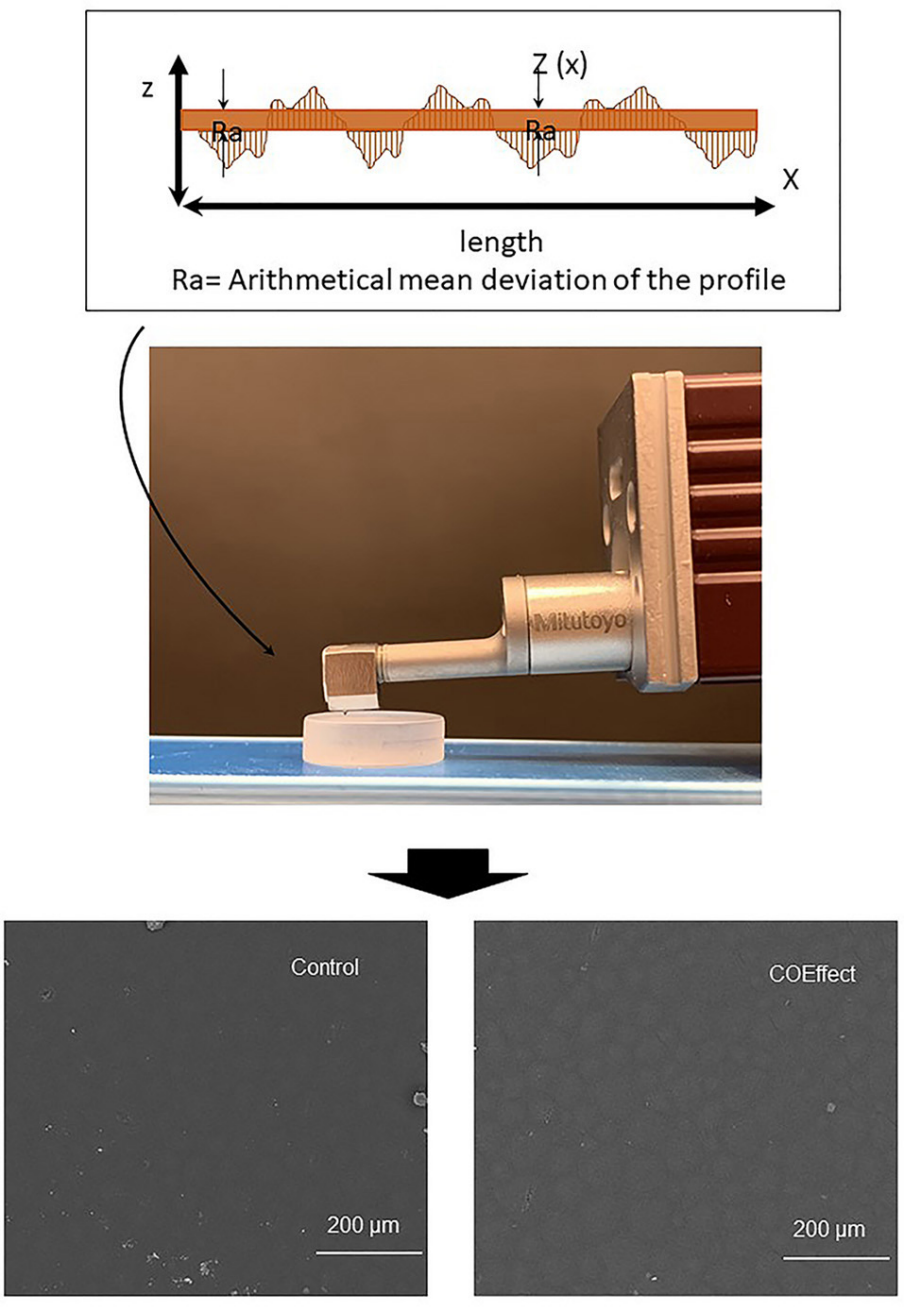
Schematic representation of roughness assessment of orthodontic acrylic resins. Illustrative scanning electron microscopy images of control (water) and one of the disinfecting solutions (Coeffect) performed to exemplify the lack of changes in the surface morphology after treatment.

The surface roughness of the orthodontic acrylic resin samples was quantified via a profilometer (Surftest SJ-301P, Mitutoyo, Tokyo, Japan). The locations for roughness measurement were randomly determined on the top surface of each sample perpendicularly, as demonstrated in Figure 1. Five measurements were obtained for each sample, and the mean value was analyzed.^21^ The selected roughness parameter was Ra, defined as the average distance from the profile to the mean line over the length of the assessment.^22^

The parameters were: the room temperature of 23°C, the diamond stylus movement speed at 0.50 mm/s, 4 mm of measuring length line, and 2.5 mm cutoff. Only one examiner performed the measurements to exclude possible errors.^23^ The roughness of each sample was calculated by the arithmetic mean of five different measurements (µm). The alteration in surface roughness (∆Ra) was attained by the difference between the roughness before and after disinfection. Moreover, illustrative scanning electron microscopy images of control (water) and one of the disinfection solutions (Coeffect) was performed to exemplify the lack of changes in the surface morphology after disinfection (Figure 1).

### Statistical analysis

The data analysis was achieved with SigmaPlot®, Version 12.0 (Systat Software, San Jose, CA, USA). Data distribution was assessed using the Shapiro–Wilk test. The intra-group differences in roughness before and after the contact with disinfectant agents were analyzed via the Wilcoxon Signed Rank test for non-parametric data or paired Student’s t-test for parametric data. Kruskal-Wallis was conducted to compare the values of the variance of roughness in percentage between disinfectant agent groups. A significance level of 0.05 was considered for all the tests.

## Results

**Figure 2:**
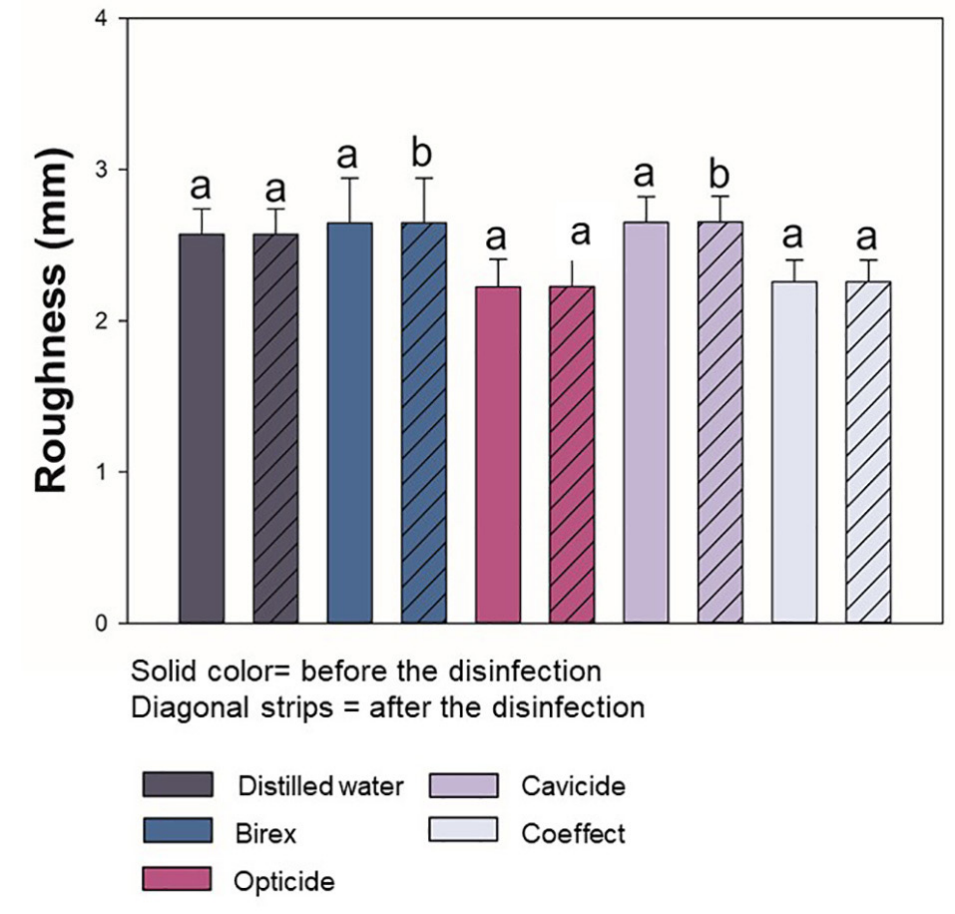
Means and standard deviations of surface roughness of acrylic resin for orthodontic appliances, respectively, subjected to disinfection with different disinfecting solutions.

Figure 2 displays the mean and standard deviation values of surface roughness for each group before and after applying disinfectant agents. The values are expressed in micrometers. The groups that were in contact with distilled water, Opti-Cide, and Coeffect did not differ in surface roughness. No significant difference was found in the paired analysis between the values “before” and” after” for distilled water, Opti-Cide, and Coeffect (P>0.05). Birex and Cavicide solutions resulted in significant differences between the initial and the final values (P<0.05).

## Discussion

This study evaluated the effect of four commercially available disinfectant agents on orthodontic acrylic resin specimens compared with distilled water as a control group. An increase in the surface roughness of dental material might contribute to microbial colonization and biofilm development on their surfaces.^24^ Chemical agents that do not change this physical property are desirable to maintain the reliability of the material used.^25^ The most commonly used material to fabricate the polymeric component of the removable orthodontic appliances is polymethylmethacrylate (PMMA). These polymers are mainly two-part systems that consist of PMMA powder beads and methylmethacrylate monomer liquid with a minute fraction of a crosslinking agent.^26^ For this type of material, the higher the roughness, the more challenging it is to remove the biofilm.^27^

In this in vitro investigation, we decided to increase the challenging scenario for PMMA material by mimicking a clinical scene where the self-curing acrylic resin is not polished. Repair and relining of removable orthodontic appliances might be necessary along the years of orthodontic treatment. When located on the internal surface of orthodontic appliances, the acrylic resin is not polished to keep the morphology of the patients’ anatomical structure. Therefore, a high roughness is observed in these areas, increasing the susceptibility to bacterial colonization.

Previous studies have shown that a roughness value of 0.2 μm is a threshold for the retention of bacteria on the surface.^28,29^ Below 0.2 μm, the surface can be judged as soft, and other physicochemical properties of the material might have a more significant role in biofilm development.^30^ The properties of orthodontic self-curing acrylic resins have been investigated in previous research after treatment with commercially available solutions. Previous reports have indicated changes in surface roughness induced by chemical agents used for disinfection, mainly solutions containing alcohol.^31,32^

Nevertheless, in these studies, the specimens were progressively polished with silicon carbide sandpaper and polishing solution, leading to a smooth surface as a baseline, without considering the non-polished areas of the orthodontic appliances. In comparison to the present study, we did not polish the specimens to mimic a situation when clinicians do not perform these steps. In this way, we could observe the effects of disinfectants even with a short period of contact in an acrylic resin with increased roughness at baseline. In contrast to previous research,^33^ the disinfectant solutions composed solely of alcohol and distilled water used as control did not modify the comparison of roughness before and after the contact. However, Birex and Cavicide, the two solutions that contained more components, such as quaternary ammonium compounds, acids, and anionic surfactants, influenced the roughness of the self-curing acrylic resins.

A stylus-type surface profilometer was utilized to quantify the roughness of acrylic resin for orthodontic purposes, obtaining the values in microns. The small variations in the vertical movement of the stylus according to the position are measured and recorded simultaneously. During the scanning, the topographical structure of the surface sample is revealed. High values from 2.23 (±0.18) µm to 2.65 (±0.17) µm were observed before treatment with some of the disinfectant agents. These values indicate that the averages between the peaks and valleys along the areas measured were high. Even with a short period of application of the solutions available in spray, paired statistical analyses showed that Birex and Cavicide solutions modified the surface compared to the control group.

Previous studies support the impact of the composition of different solutions on acrylic resin materials.^34,35^ Basavarajappa et al^35^ demonstrated that auto-polymerized polymers are prone to surface crazing and dissolution by ethanol-based disinfectants. Machado et al^36^ showed that disinfection using sodium perborate solution and microwave disinfection did not compromise the hardness of acrylic resins. However, these methods might unfavorably increase the surface roughness, and the effect appears to be material-dependent. Under the same perspective, Matos et al^37^ highlighted the potentially detrimental impact of disinfection solutions on the bond strength of microwave-cured acrylic resins.

In general, it seems challenging to determine the ideal antimicrobial solution that can disinfect the acrylic resin without any long-term effects on the mechanical and physical properties of the material. Currently, there is no specific agent or specific protocol to be recommended for disinfecting acrylic resin materials, especially with the considerable concern related to materials’ properties.^13^ Several studies reported some changes related to the strength, hardness, color, and roughness of the acrylic resin materials following disinfection.^9,11,38^ Another recent study found that the short-term acrylic resin disinfection did not affect the flexural strength, surface roughness, and the color of different commercially available orthodontic acrylic resins.^33^

These uncertain outcomes could be related to the type of the material, the type of disinfectant, the followed protocol, and disinfection duration. Future studies must emphasize the need to examine both short- and long-term disinfection and its diverse effects on acrylic resin materials’ characteristics. With all these limitations in the literature, it is hard to recommend or establish a specific protocol to disinfect orthodontic acrylic resins.

Despite the statistical differences observed, the values of variance in percentage were minimal, reflecting the decreased values and absolute results. The little differences generated could be jeopardized by the time of contact with the solutions. The limitations of this research include the lack of intervals between the use of the disinfectant agents to mimic how patients would use the sprays on orthodontics devices. Along with the treatment of the resins with the disinfecting agents, we did not expose the samples to artificial saliva or water for long periods, which could increase the softening of the material and decrease roughness. Further studies could be performed to mimic this clinical situation and account for factors such as the presence of saliva. Moreover, other properties, such as hardness and flexural strength, could characterize the in-depth behavior of the material after treatment.

## Conclusion

Orthodontic appliances have retentive areas prone to biofilm retention on their surfaces. Disinfecting spray agents are convenient and help with the compliance of orthodontic patients. We observed that even with a short period of contact with the disinfectant agents, the orthodontic self-curing acrylic resins exhibited changes in surface roughness. However, with minimal variance in comparison to distilled water. The use of disinfectant solutions is unlikely to damage the surface of orthodontic acrylic resins. Oral care providers should exercise caution in interpreting and implementing clinically meaningful changes in their evidence-based approach regarding potential material damage by the disinfection sprays.

## Acknowledgments

This study was financed in part by the Coordenação de Aperfeiçoamento de Pessoal de Nível Superior, Brasil (CAPES) - Finance Code 001 – scholarship of IMG.

## Author Contributions

Conceptualization: KLH, LDP, MAM. Experimental work: KLH and MAM. Original draft preparation: IMG, AAB, and MAM. Funding acquisition: LDP. Draft revision and editing: FMC and MAM. All the authors have read and agreed to the published version of the manuscript.

## Competing Interests

The authors declare no conflict(s) of interest related to the publication of this work.

## Funding

This study was funded by GC America, Alsip, IL, USA.

## Ethics Approval

Not Applicable.
